# Hypothalamic volume in pedophilia with or without child sexual offense

**DOI:** 10.1007/s00406-022-01501-w

**Published:** 2022-11-12

**Authors:** Melanie Storch, Maria Kanthack, Till Amelung, Klaus M. Beier, Tillmann H. C. Krueger, Christopher Sinke, Henrik Walter, Martin Walter, Boris Schiffer, Stephanie Schindler, Peter Schoenknecht

**Affiliations:** 1 Department of Psychiatry and Psychotherapy, University Hospital Leipzig, Semmelweisstr. 10, 04103 Leipzig, Germany; 2 Department of Biology, University of Leipzig, 04103 Leipzig, Germany; 3 Institute of Sexology and Sexual Medicine, Charité-Universitätsmedizin Berlin, Corporate Member of Freie Universität Berlin, Humboldt-Universität Zu Berlin, and Berlin Institute of Health, 10117 Berlin, Germany; 4 Department of Psychiatry and Neurosciences, Charité-Universitätsmedizin Berlin, Corporate Member of Freie Universität Berlin, Humboldt-Universität Zu Berlin, and Berlin Institute of Health, 10117 Berlin, Germany; 5 Division of Clinical Psychology and Sexual Medicine, Department of Psychiatry, Social Psychiatry and Psychotherapy, Hannover Medical School, 30625 Hanover, Germany; 6 Center for Systems Neuroscience Hannover, Hanover, Germany; 7 Division of Mind and Brain Research, Department of Psychiatry and Psychotherapy, CCM, Charité-Universitätsmedizin Berlin, Corporate Member of Freie Universität Berlin, Humboldt-Universität Zu Berlin, and Berlin Institute of Health, 10117 Berlin, Germany; 8 Department of Psychiatry, Otto-Von Guericke-University Magdeburg, 39106 Magdeburg, Germany; 9 Department of Psychiatry and Psychotherapy, University Hospital Jena, 07743 Jena, Germany; 10 Division of Forensic Psychiatry, Department of Psychiatry, Psychotherapy and Preventive Medicine, Ruhr University Bochum, LWL University Hospital, 44791 Bochum, Germany; 11 Medical Faculty, Department of Psychiatry and Psychotherapy, University Hospital Leipzig, 04103 Leipzig, Germany; 12 Out-Patient Department for Sexual-Therapeutic Prevention and Forensic Psychiatry, University Hospital Leipzig, 04103 Leipzig, Germany; 13 Academic Saxon State Hospital Arnsdorf, 01477 Arnsdorf, Germany

**Keywords:** Pedophilia, Child sexual offending, Hypothalamus, Volume, Human, Magnetic resonance imaging

## Abstract

The hypothalamus regulates sexual behavior and is simultaneously associated with aggression and violence. Consequently, this brain region is relevant in research of pedophilia and child sexual offenses (CSO). The distinction between these two phenomena is of great importance and was the object of consideration of this study. We analyzed exclusively men, including 73 pedophilic offenders who committed CSO, an equal number of people with pedophilia but without such offenses, and 133 non-pedophilic, non-offending subjects who formed the control group. All data were collected in a multicenter in vivo study and analyzed using a semi-automated segmentation algorithm for 3-Tesla magnetic resonance images. Men with pedophilia who committed CSO on average had a 47 mm^3^ smaller hypothalamus per side than people without committed CSO. This effect was driven by both the group of non-offending people with pedophilia and the control group. By contrast, the exploratory comparison of pedophilic persons without CSO with the control group showed no significant difference. The present study demonstrates a deviant hypothalamic structure as a neurobiological correlate of CSO in pedophiles, but not in people with pedophilia who have not committed CSO. Thus, it strengthens the argument to distinguish between sexual offending and paraphilic sexual preferences.

**Supplementary Information:**

The online version contains supplementary material available at 10.1007/s00406-022-01501-w.

## Introduction

The experience of child sexual abuse is associated with immediate physical and emotional effects, as well as elevated long-term risks of serious psychiatric, psychosocial, and physical health outcomes [1, 2] and its global prevalence rate is a notable 12.7% [3]. Research on the neurobiological underpinnings of child sexual offense (CSO) and pedophilia is crucial for our understanding of their etiology and treatment, a cornerstone of CSO prevention [4]. Victims, survivors, and perpetrators alike are certain to benefit from research and clear information.

Among the most important keys to understand pedophilia and CSO is the awareness that they are separable phenomena. One does not necessarily imply the other and both can occur independently of one another or together. Only 40–50% of all child sexual offenders show clinical signs of pedophilia [5] and “only” about 43% of all people with pedophilia commit sexual offenses against children [6]. Pedophilia could therefore be considered a risk factor for committing a CSO, but they are not equivalent. Ignoring this distinction is a fundamental problem of several studies in the past, which inferred pedophilia from CSO without diagnosing it independently [7].

A plausible opinion about delinquent sexual behavior is that it has its biological foundations in the network of brain regions that regulate sexual functions. First and foremost among these is the hypothalamus [8, 9], which is the focus of this report. Our current understanding of sexual arousal is that the hypothalamus not only affects autonomic and neuroendocrine functions but also motivational processes [7, 10–12]. Intriguingly, the hypothalamus is also a part of the neural circuit of aggressive behavior and violence [13–17]. Both aspects motivate our large-scaled study of the macrostructure of the hypothalamus in CSO.

Three single-case reports suggest that hypothalamic damage, which was caused by multiple sclerosis, glioma, or clivus chordoma, is associated with CSO [18–20]. This association does not exclude other cases of CSO following brain injuries which did not show hypothalamic damage [21]. Further evidence for structural abnormalities of the hypothalamus in CSO is provided by quantitative imaging studies. A voxel-based morphometry analysis of 15 pedophilic males who committed CSO revealed a bilateral reduction of gray matter density in the hypothalamus [22, but see also 23–26]. In addition, Walter et al. [27] reported reduced activity in the hypothalamus during sexual arousal in people with pedophilia who committed CSO compared to healthy controls. These data suggest an association between abnormal hypothalamus structure and function and CSO or pedophilia.

This effect is more likely driven by CSO-related dysfunctions than by pedophilia. Several studies indicate that hypothalamic–pituitary–adrenal (HPA) axis activity is inversely related to violent aggression [28–31]. Reduced baseline plasma cortisol, an indicator for HPA axis activity, in sexually offending pedophilic men [32] supports this assumption. Additionally, data analyses suggest that testosterone which has long been suspected to be the major culprit in aggression and violent crime [33–37] requires reduced cortisol levels to work its effects [38, 39]. This further strengthens the glucocorticoid deficit hypothesis according to which HPA axis hypofunction is associated with violent aggression and CSO, presumably via epigenetic changes mainly in the prefrontal cortex [31, 32]. A hypothalamic volume reduction may be a structural marker of a downregulated HPA axis.

However, people with pedophilia who have not committed CSO have been underrepresented in past research. The few existing studies of non-offending pedophilic men and offending pedophilic men concluded that structural changes in the brain [25, 40], (epi-)genetic alterations of the androgen [37] and the serotonergic [41] system as well as executive dysfunctions [42] are more likely related to CSO and not to pedophilia alone, as was long believed.

Summarized, the data available to date suggest that CSO rather than sexual preference is crucial to manifest brain alterations in vivo. Previous findings of abnormalities in pedophilia are limited due to diagnostic imprecision, that is, the inference of pedophilia from CSO. We therefore hypothesized a CSO-related reduced hypothalamic volume in pedophilic offenders as compared to both non-offending groups combined as well as both the control group and people with pedophilia without a history of CSO, separately. Subsequently, we assumed no significant differences between hypothalamic volumes of the control group and non-offending people with pedophilia.

## Method

### Participants

Participants were recruited in the context of the multicenter study “Neural Mechanisms Underlying Pedophilia and Sexual Offending against Children” (NeMUP, www.nemup.de). The Project was a collaboration of five sites in Germany (Charité-Universitätsmedizin Berlin, Hannover Medical School, Universities of Duisburg-Essen, Kiel, and Magdeburg) and funded by the German Federal Ministry of Research and Education. Subjects were either participants of the “Prevention Project Dunkelfeld” (www.dont-offend.org) [43], where self-identified people with pedophilia are offered anonymous therapy or they were recruited via prisons, online advertisements, forum posts, and mailing lists, or during fulfilment of a suspended sentence. For a more detailed description of the total NeMUP sample and its recruitment procedures please refer to Gerwinn et al. [44]. The all-male sample (Table 1) consisted of 73 people with pedophilia with a history of sexual offense against children (P + CSO), 73 people with pedophilia without such offense (P-CSO), and 133 teleiophilic men without committing CSO. The latter was the control group.

**Table 1:** Sociodemographic and clinical characteristics of all study groups

Characteristics	P + CSO	P-CSO	Controls	Test statistics and *p*-values
*n*	73	73	133	
Age in years, *M* (SD)	39.99 (9.17)	34.78 (9.35)	33.35 (10.10)	*H*_2_ = 24.42, *p* < 0.001***
ICV in mm^3^, *M* (SD)	1,563,719.23 (131,651.05)	1,626,388.32 (120,795.78)	1,625,977.95 (142,135.48)	*H*_2_ = 9.86, *p* = 0.007**
Handedness quotient (EHI), *M* (SD)	68.49 (47.56)	66.63 (49.54)	65.90 (56.35)	*H*_2_ = 0.49, *p* = 0.783
Intelligence (WAIS-score), *M *(SD)				
Verbal	93.37 (18.48)	106.50 (18.35)	103.71 (20.32)	*H*_2_ = 19.29, *p* < 0.001***
Performance	104.01 (18.74)	108.34 (18.22)	105.89 (18.54)	*H*_2_ = 2.20, *p* = 0.333
General ability	98.96 (19.28)	108.00 (17.15)	105.75 (18.99)	*H*_2_ = 11.54, *p* = 0.003**
Mental disorders (DSM-IV, lifetime) in %	63.9	61.6	25.6	*χ*^*2*^_2, *n*=277_ = 38.28, *p* < 0.001***
Personality disorders (DSM-IV, lifetime) in %	37.1	41.1	5.3	*χ*^*2*^_2, *n*=275_ = 46.03, *p* < 0.001***

Group comparisons are reported with degrees of freedom as subscripts

*χ*^*2*^ Chi-square test, *DSM-IV* Diagnostic and Statistical Manual of Mental Disorders—fourth edition, *EHI* Edinburgh Handedness Inventory, *H* Kruskal–Wallis *H*-test, *ICV* intracranial volume, *M* mean, *n* number of participants, *P + CSO* pedophilic persons with a history of sexual offense against children, *P-CSO* non-offending people with pedophilia, *SD* standard deviation, *WAIS* Wechsler Adult Intelligence Scale, **p < 0.01, *** *p* < 0.001

Pedophilic subjects had to fulfill the diagnostic criteria for pedophilia according to International Statistical Classification of Diseases and Related Health Problems (ICD-10) [45]. This encompasses people who are sexually attracted to children before puberty and in early pubescent stages. In this study, CSO indicates that the person has committed at least one hands-on offense against a child under the age of 14. In detail, this means that the naked body or genitals of a child have been touched or have been manually or orally manipulated with the intention of sexually stimulating oneself, penetrating the child vaginally or anally, or causing the child to touch the offender’s genitals or penetrate him.

Exclusion criteria were any acute psychotic or substance use disorder, neurological disorders, somatic illnesses, contraindications for magnetic resonance imaging (MRI), psychotropic medication, or other psychopharmacological treatment that affects sexual functioning.

The ethics committees of all research sites involved in the NeMUP project approved the study and all participants gave their written consent before participating.

### Measures

To asses Axis I and II disorders, the Structured Clinical Interview (SCID) for DSM-IV-TR (Diagnostic and Statistical Manual of Mental Disorders—fourth edition) [46] was applied by trained psychologists. Additionally, the sexual biography was obtained by a semi structured interview. This included sexual interests, sexual offenses, general offenses, current sexual functioning, masturbation, and intercourse frequencies, ejacularche, onset of masturbation, as well as the lifetime and current consumption of media showing sexual violence against children or indicative images (images of children in underwear, bathing suits, and similar). Sexual orientation, fantasies, and age preferences were verified using a modified version of the Kinsey Scale for developmental stages [47] in combination with the Tanner stages I-V [48].

An adapted version of the German Edinburgh Handedness Inventory (EHI) [49], consisting of 10 items, was used to calculate the handedness quotient. The intelligence of the participants was measured with the German version of the third edition of the Wechsler Adult Intelligence Scale (WAIS) [50]. The subtests Vocabulary and Similarities were used to assess verbal intelligence whereas Block Design and Matrix Reasoning were used to calculate the performance IQ.

### Image acquisition and preprocessing

Structural T1-weighted MR images were acquired at five separate 3-Tesla MRI scanners (two Siemens Skyra, two Siemens Trio, and one Phillips Achieva). 32 channel head coils were used with a magnetization-prepared, rapid gradient-echo sequence with an isotropic spatial resolution of 1 mm^3^ (MPRAGE [51]; slices = 192, repetition time = 2500 ms, echo time = 4.33 ms, flip angle = 7°).

Preprocessing of the MR images was performed analogously to the method described in our published hypothalamus segmentation algorithm [52]. In short, the scans were aligned with the coordinate system of the reference atlas [53], using the LIPSIA 2.2 software [54] and shifted linear interpolation [55]. All T1-weighted scans were subsequently skullstripped with the FSL software version 5.0.9 [56] and were stored in NIfTI format. To estimate the intracranial volume (ICV), the number of all voxels with intensities above zero was determined after skull-stripping.

The algorithm for the hypothalamus segmentation [52] requires grey matter tissue probability maps (GM-TPMs) which were created with a very light bias regularization and the Segment tool in the SPM12 software package (Statistical Parametric Mapping) [57] using MATLAB R2018a (www.mathworks.com, Mathworks Inc., USA). The MRI scans were equally distributed among two raters by stratified randomization with respect to scanner location and study group. The software RITA (Version 1.5) [58] was used for the permuted block randomization within strata [59]. The scans were displayed either unflipped or right-left flipped (done with FSL software) to the two raters which were blinded with respect to group membership or hemispheres, since the segmentations were done on the left side of the computer screen only.

### Hypothalamus segmentation

Unilateral segmentation of the hypothalamus according to the semi-automated volumetry algorithm [52] was performed with the MeVisLab software (Medical Image Processing and Visualization 2.4, Frauenhofer Institut, Bremen, Germany, www.mevislab.de) on 24" LED monitors. To achieve comparable contrast all scans were presented with values calculated for each scan separately (window = 1.5 × median of intensities; center = window/2) as recommended by Wendt [60]. Triplanar view was used for all of the following segmentation steps. The T1-weighted 3 T MRI scans were overlaid with the GM-TPMs which were displayed in green (red–green–blue color code: 50, 198, 47) with 80% opacity. The algorithm defines different boundaries for each of four subsections of the hypothalamus (preoptic, intermediate—superior, intermediate—inferior, and posterior). Firstly, boundaries of regions of interests (ROIs) were set unilaterally in coronal view using anatomical landmarks specified in the algorithm. Secondly, the four ROIs were reviewed and, if necessary, corrected in the horizontal and sagittal views, to achieve anatomical plausibility. Thirdly and lastly, a seed voxel was placed within each of the four sections, designating the starting point for the software’s seed growing algorithm. The software then automatically calculated which voxels were to be included or excluded in the segmentation up to the edge of the ROIs. For this automated step, the previously created GM-TPMs were particularly crucial. They assign a value between 0 and 1 to each voxel, indicating its probability of belonging to gray matter. After verification if also suitable for our data, we defined the thresholds for the four sections of the hypothalamus to achieve an anatomically correct segmentation using the values of the algorithm. Finally, the segmentations of the individual ROIs were combined to form the segmentation of the entire unilateral hypothalamus.

### Reliability

Before volumetry of all subjects, 20 MRI scans were selected for reliability testing to verify measurement accuracy of the two trained raters. Mean age and mean ICV of the reliability sample (age in years, *M* (SD): 36.2 (10.1), ICV in mm^3^, *M* (SD): 1,618,214 (183,496)) were representative for the full sample (age in years, *M* (SD): 36.0 (10.4), ICV in mm^3^, *M* (SD): 1,609,725 (140,284)) and equal distribution of scanner location and group membership was observed during the selection procedure. Blinding and segmentation of the 40 unilateral hypothalami of the reliability test were performed exactly as described above for the entire sample.

The interrater reliability of the two raters was calculated in terms of the intraclass-correlation-coefficient (ICC) with the absolute agreement model [61] as it also punishes mean differences and this may be regarded as very strict. The two raters achieved ICC_39,35_ = 0.95 and this can be considered an excellent reliability [61]. Dice-coefficient [62], which describes the degree of spatial overlap between segmentations of the two raters, was 94.7%.

### Statistics

All statistical analyses were performed using the software SPSS (IBM Corp. released 2019, IBM SPSS Statistics for Windows, version 26.0. Armonk, NY: IBM Corp.). Tests were performed two-tailed and significance was assumed for *p*-values < 0.05. The volume of the hypothalamus in mm^3^ was calculated as the number of segmented voxels. Normal distribution and homogeneity of variances was tested using Shapiro–Wilk tests and Levene tests, respectively. We compared the sociodemographic and clinical characteristics of all groups using Kruskal–Wallis *H*-tests and Chi-square tests.

Group comparisons followed a stepwise procedure. We started with a multivariate analysis of variance (MANOVA) for the left and right hypothalamus volumes as dependent variables and group membership as independent variable. Roy's largest root was evaluated, which was shown to have the best statistical properties for data with normal distribution, homogeneous variances, and two dependent variables [63]. We then added the potential confounders age and ICV that correlated with the hypothalamus volume (left hypothalamus: age: *r*_277_ = -0.33, *p* < 0.001, ICV: *r*_277_ = 0.50, *p* < 0.001; right hypothalamus: age: *r*_277_ = -0.30 *p* < 0.001, ICV: *r*_277_ = 0.47, *p* < 0.001) and differed significantly between the study groups (age: *H*_2_ = 24.42, *p* < 0.001; ICV: *H*_2_ = 9.86, *p* = 0.007). The covariates were added first individually and then simultaneously, as these did not correlate with each other (*r*_277_ = -0.07, *p* = 0.217). Significant MAN(C)OVAs were followed by univariate analyses of (co)variance (AN(C)OVAs).

The univariate global comparisons were complemented by simple contrasts comparing all subjects which have committed CSO with all non-offenders (-CSO) according to our first hypothesis. The second and third hypotheses were tested using simple contrasts of P + CSO compared to P-CSO and P + CSO compared to control subjects. Finally, for exploratory purposes, the non-offending pedophilia group was compared with the control group, since testing for equivalence is not feasible with a sample as small as ours [64]. We report significance levels after correction for bilateral testing using the Bonferroni–Holm procedure. Five unilateral hypothalamus volumes were identified as statistical outliers as defined by their group-wise z-scores |*z*|≥ 2.58 (left hypothalamus: P + CSO: 950 mm^3^, *z* = 2.59 and 957 mm^3^, *z* = 2.68; right hypothalamus: P + CSO: 548 mm^3^, *z* = -2.62 and 936 mm^3^, *z* = 2.87, control group: 974 mm^3^, *z* = 2.64). Sensitivity analyses after outlier exclusion were performed to evaluate the robustness of all inferential statistics.

## Results

### Sample characteristics

As shown in Table 1, the three study groups did not differ with respect to handedness and WAIS performance IQ. However, there was a significant group difference for age, ICV, and verbal or general ability WAIS scores, with the P + CSO group being the oldest, the one with the smallest ICV, and the one with the lowest WAIS scores. The groups also differed significantly with regard to DSM-IV mental and personality disorders, with proportionally fewer people in the control group having diagnoses in both categories.

### Global group comparisons and contrasts

**Fig. 1:**
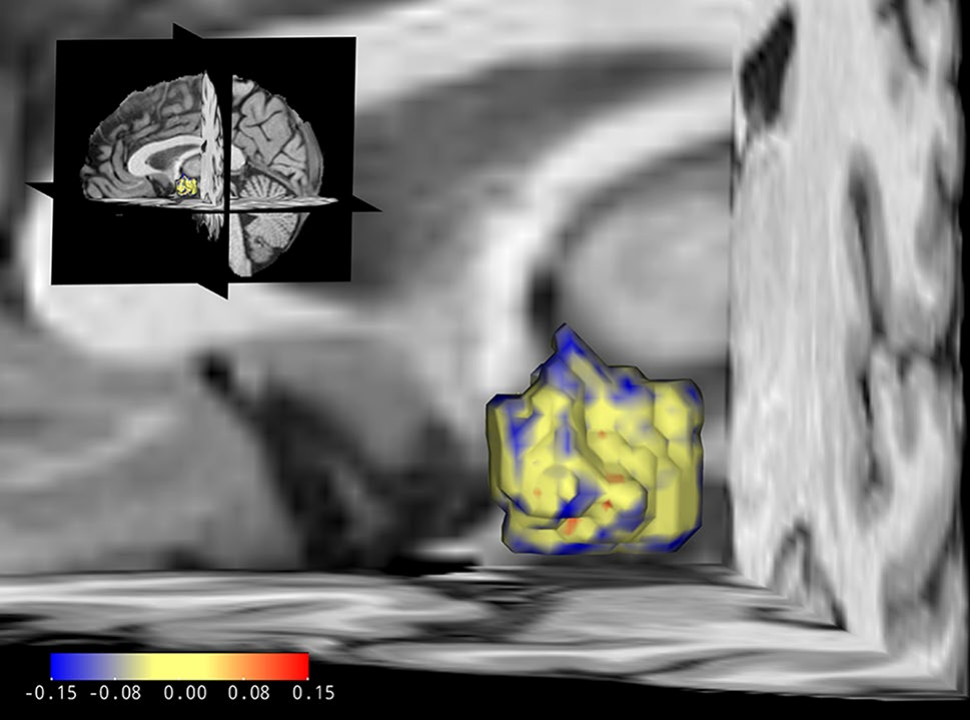
Left hypothalamus in a 3D reconstruction with colored local volume differences. Differences between absolute hypothalamic volumes of child sexual offending participants (CSO, *n* = 73) and non-offenders (-CSO, *n* = 206) are color coded, as shown in the map at the bottom left. Blue color indicates local volume reductions of CSO compared to -CSO and red locally larger volumes in CSO. The displayed sample hypothalamus was created for this purpose by linear registration of all left hypothalamus segmentations. Probability masks for the two groups were thresholded at 1/3

**Fig. 2:**
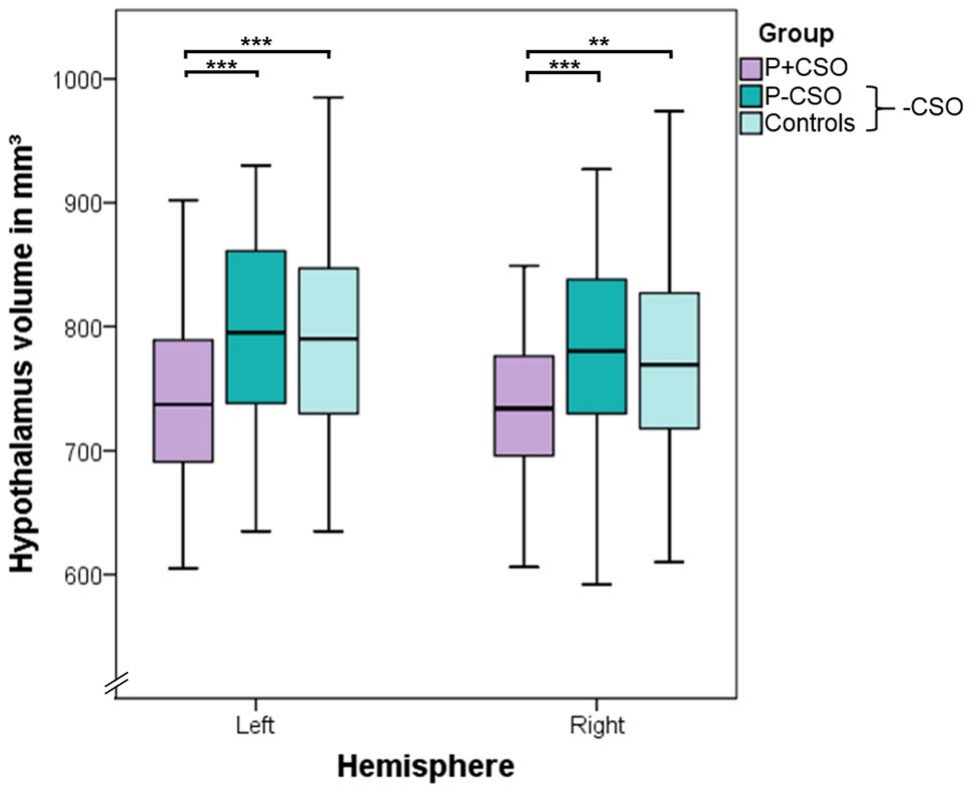
Hypothalamic volume reduction in pedophilic offenders*.* Statistical results of Absolute volumes are displayed, outliers have been hidden, whiskers denote 1.5 × interquartile range. Abbreviations: *CSO* committed child sexual offense, *-CSO* no committed child sexual offense, *P + CSO* pedophilic persons with a history of sexual offense against children, *P-CSO* non-offending people with pedophilia, **p < 0.01, ***p < 0.001

For statistics of our hypothalamus volumes and group comparisons regarding these please see Tables 2 and 3, respectively. Our first analysis step, a MANOVA of absolute hypothalamic volumes across all three groups indicated a statistically significant difference [*F*_2,276_ = 12.26, *p* < 0.001, partial *η*^2^ = 0.082 (90%-CI [0.034, 0.133]), Roy's largest root = 0.089]. This was confirmed in the univariate group comparisons and after Bonferroni–Holm correction (left hypothalamus: *F*_2,276_ = 11.30, *p* < 0.001, right hypothalamus: *F*_2,276_ = 9.21, *p* < 0.001). To test our first hypothesis of reduced hypothalamus volume in CSO, the participants without CSO (P-CSO and controls) were grouped together and compared with P + CSO (Table 3). These a priori planned contrasts yielded significant differences for the left (Fig. 1; *M*_Diff_ = −51.41, 95%-CI [−72.68, −30.13], *p* < 0.001) and right (*M*_Diff_ = −42.15, 95%-CI [−62.19, −22.11], *p* < 0.001) absolute hypothalamus volumes. To test our second and third hypotheses of a volume reduction in P + CSO relative to the pedophilic non-offending and the control group, we complemented the analysis with two corresponding contrasts (P + CSO versus P-CSO and P + CSO versus controls). Both pairwise comparisons revealed significant differences in absolute left and right hypothalamus volumes in the expected direction (Fig. 2).

**Table 2:** Hypothalamus volumes in mm^3^

Groups	*n*	Absolute volumes		*n* ^OE^	Corrected for ICV and age ^OE^	
		Left	Right		Left	Right
P + CSO, *M* (SD)	73	740.70 (80.84)	733.18 (70.70)	70	757.95 (68.27)	749.95 (65.37)
P-CSO, *M* (SD)	73	793.77 (80.59)	783.19 (74.36)	73	788.08 (65.75)	778.34 (62.95)
Controls, *M* (SD)	133	791.20 (78.10)	771.02 (76.99)	132	782.10 (66.46)	762.27 (63.64)

Absolute and with ANCOVA corrected mean hypothalamus volumes

*ICV* intracranial volume, *M* mean, *n* number of participants, *OE* statistical outlier excluded, *P + CSO* pedophilic persons with a history of sexual offense against children, *P-CSO* non-offending people with pedophilia, *SD* standard deviation

**Table 3:** (M)AN(C)OVAs and contrast testing results regarding hypothalamus volume

Group comparisons	*n*	Absolute volumes		Corrected for ICV		Corrected for age		Corrected for ICV and age	
Multivariate	279	*F*_2, 276_ = 12.26, *p* < 0.001***		*F*_2, 275_ = 6.78, *p* = 0.001***		*F*_2, 275_ = 6.93, *p* = 0.001***		*F*_2, 274_ = 3.13, *p* = 0.045*	
	275 ^OE^	*F*_2, 272_ = 14.39, *p* < 0.001***		*F*_2, 271_ = 8.83, *p* < 0.001***		*F*_2, 271_ = 8.80, *p* < 0.001***		*F*_2, 270_ = 4.61, *p* = 0.011*	
		Left	Right	Left	Right	Left	Right	Left	Right
Univariate	279	*F*_2, 276_ = 11.30, *p* < 0.001***	*F*_2, 276_ = 9.21, *p* < 0.001***	*F*_2, 275_ = 6.08, *p* = 0.006**	*F*_2, 275_ = 4.89, *p* = 0.008**	*F*_2, 275_ = 6.20, *p* = 0.004**	*F*_2, 275_ = 5.43, *p* = 0.005**	*F*_2, 274_ = 2.51, *p* = 0.146	*F*_2, 274_ = 2.65, *p* = 0.146
	275 ^OE^	*F*_2 272_ = 13.51, *p* < 0.001***	*F*_2, 272_ = 10.19, *p* < 0.001***	*F*_2, 271_ = 8.13, *p* < 0.001***	*F*_2, 271_ = 5.91, *p* = 0.003**	*F*_2, 271_ = 8.09, *p* < 0.001***	*F*_2, 271_ = 6.45, *p* = 0.002**	*F*_2, 270_ = 3.99, *p* = 0.040*	*F*_2, 270_ = 3.52, *p* = 0.040*
Contrasts		Left (*M*_Diff_, [95%-CI])	Right (*M*_Diff_, [95%-CI])	Left (*M*_Diff_, [95%-CI])	Right (*M*_Diff_, [95%-CI])	Left (*M*_Diff_, [95%-CI])	Right (*M*_Diff_, [95%-CI])	Left (*M*_Diff_, [95%-CI])	Right (*M*_Diff_, [95%-CI])
CSO vs. -CSO	279	*−*51.41, [−72.68, −30.13], *p* < 0.001***	−42.15, [−62.19, −22.11], *p* < 0.001***	−33.93, [−43.11, −14.76], *p* = 0.001**	−26.83, [−45.23, −8.42], *p* = 0.004**	−37.71, [−59.01, −16.42], *p* = 0.001**	−30.51, [−50.72, −10.30], *p* = 0.003**	NA	NA
	275 ^OE^	−55.70, [−55.70, −34.61], *p* < 0.001***	−43.08, [−62.72, −23.43], *p* < 0.001***	−39.03, [−58.12, −19.95], *p* < 0.001***	−28.77, [−46.91, −10.63], *p* = 0.002**	−42.61, [−63.65, −21.56], *p* < 0.001***	−32.21, [−51.98, −12.45], *p* = 0.001**	−26.37, [−45.20, −7.54], *p* = 0.012*	−18.28, [−36.39, −0.16], *p* = 0.048*
P + CSO vs. P-CSO	146	−53.07, [−78.96, −27.96], *p* < 0.001***	−50.01, [−74.36, −25.67], *p* < 0.001***	−35.52, [−58.71, −12.33], *p* = 0.004**	−34.63, [−56.83, −12.42], *p* = 0.004**	−41.37, [−66.79, −15.94], *p* = 0.002**	−39.93, [−63.98, −15.87], *p* = 0.002**	NA	NA
	143 ^OE^	−57.75, [−83.29, −32.22], *p* < 0.001***	−51.91, [−75.62, −28.20], *p* < 0.001***	−40.97, [−68.93, −18.01], *p* = 0.001**	−37.51, [−59.26, −15.76], *p* = 0.001**	−46.52, [−71.55, −21.50],*p* < .001***	−42.45, [−65.86, −19.04],*p* < .001***	−30.14, [−52.42, −7.86], *p* = 0.016*	−28.39, [−49.72, −7.06], *p* = 0.016*
P + CSO vs. controls	206	−50.50, [−73.29, −27.71], *p* < 0.001***	−37.84, [−59.26, −16.42], *p* = 0.001**	−33.06, [−53.55, −12.58], *p* = 0.004**	−22.55, [−42.17, −2.84], *p* = 0.024*	−35.87, [−58.40, −12.74], *p* = .004**	−24.97, [−46.56, −3.37], ***p*** = .024*	NA	NA
	202 ^OE^	−54.57, [−77.14, −32.00], *p* < 0.001***	−38.19, [−59.15, −17.23], *p* < 0.001***	−37.96, [−58.32, −17.60], *p* < 0.001***	−23.94, [−43.23, −4.65], *p* = 0.015**	−40.29, [−62.82, −17.77],*p* = .001**	−26.17, [−47.24, −5.10], *p* = .015*	−24.15, [−44.26, −4.05], *p* = 0.038*	−12.32, [−31.57, 6.93], *p* = 0.209

Group comparisons are reported with degrees of freedom as subscripts.* CSO* committed child sexual offense,* -CSO * no committed child sexual offense, *ICV* intracranial volume, *M*_*Diff*_ mean difference, *n* number of participants, *NA* not applicable,* OE* statistical outlier excluded,* P + CSO* pedophilic persons with a history of sexual offense against children, *P-CSO* non-offending people with pedophilia, *vs.* versus, *95%-CI* 95% Confidence Interval for Difference, * *p* < .05, ** *p* < .01, *** *p* < .001

Results of global multivariate and univariate group comparisons and contrasts remained stable when corrected for ICV or age and in each case after excluding the outliers. The multivariate group comparison was still significant under simultaneous correction for age and ICV (*F*_2,274_ = 3.13, *p* = 0.045, partial *η*^2^ = 0.022 (90%-CI [0.000, 0.055]), Roy's largest root = 0.023). No effect for the group factor initially existed in the univariate analysis of the two hypothalamus volumes when corrected for age and ICV simultaneously (left hypothalamus: *F*_2,274_ = 2.51, *p* = 0.083, right hypothalamus: *F*_2,274_ = 2.65, *p* = 0.073). However, it emerged when we excluded the outliers and remained significant even after correcting for multiple comparisons (left hypothalamus: *F*_2,270_ = 3.99, *p* = 0.020, right hypothalamus: *F*_2,270_ = 3.52, *p* = 0.031). In this final analysis step, under simultaneous correction for the two confounders age and ICV and after excluding of outliers, our first hypothesis was confirmed again (planed contrasts CSO versus -CSO: left hypothalamus: *M*_Diff_ = −26.37, 95%-CI [−45.20, −7.54], *p* = 0.006, right hypothalamus: *M*_Diff_ = −18.28, 95%−CI [−36.39, −0.16], *p* = 0.048). The effects for the second hypothesis were replicated (P + CSO versus P-CSO: left hypothalamus: *M*_Diff_ = −30.14, 95%-CI [−52.42, −7.86], *p* = 0.008, right hypothalamus: *M*_Diff_ = −28.39, 95%-CI [−49.72, −7.06], *p* = 0.009) and those for the third hypothesis to a major extent (P + CSO versus controls: not on the right but on the left side of the hypothalamus: *M*_Diff_ = −24.15, 95%-CI [−44.26, −4.05], *p* = 0.019).

None of the four analysis steps (absolute volumes and controlling for age, ICV, and age and ICV) could detect a significant difference in the exploratory comparisons of the left and right hypothalamus volumes of the P-CSO group with controls (Table S1, supplementary material, to illustrate two: absolute volume: left hypothalamus: *M*_Diff_ = 2.57, 95%-CI [−20.22, 25.36], *p* = 0.824, right hypothalamus: *M*_Diff_ = 12.18, 95%-CI [−9.25, 33.60], *p* = 0.528).

## Discussion

### Child sexual offenders show hypothalamic volume reduction

In agreement with our first hypothesis of a reduction of hypothalamus volume in CSO, we observed a hypothalamic reduction in persons with pedophilia who committed CSO. The effect was evident in both hypothalamic hemispheres in absolute volumes and after correction for the two confounders ICV and age separately and simultaneously after outlier exclusion. Following contrasts confirmed our hypotheses that the effect was driven by the control as well as the P-CSO group. In our study CSO is related to hypothalamic volume reduction in pedophilic men and this effect is driven by the control group as well as the pedophilic non-offender group.

Significance was initially lost after simultaneous correction for ICV and age in the global univariate group comparisons of hypothalamus volumes and reemerged after excluding the statistical outliers. This suggests suppression effects due to multicollinearity and a sensitivity of the parametric models to non-normal outliers. The statistical outliers could not be explained by variations in clinical characteristics, measurement, or sampling. To account for the known sensitivity of parametric tests to outliers, we reported results before and after excluding statistical outliers for the purpose of transparency and reliability.

Since one contrast of volume reduction was not significant in the right hypothalamus, we may expect that the effect is more prominent on the left side. This would be consistent with the findings of a volume reduction of the right amygdala [24] and a functional connectivity between the right amygdala and the left hypothalamus in pedophilic offenders [65]. Blinding the rates to the hemispheres minimized the likelihood that the algorithm was applied differently.

Pedophilic participants both with and without histories of committed CSO consumed material depicting child sexual abuse, indicating the alteration in hypothalamic structure appears to be associated with implementation of CSO at the behavioral level. Our findings corroborate studies highlighting the hypothalamus and its subsequent cascades and regulatory mechanisms in violence [13–17]. Furthermore, our results are consistent with previous studies that focused on sexual violence against children and showed an activity reduction in the hypothalamus in pedophilic offenders [27] and acquired pedophilia and CSO after hypothalamic damages [18–20]. More precisely our findings possibly confirm our initial assumption that a reduced hypothalamic volume may indicate a reduced HPA axis activity. The deficit of glucocorticoids, induced by the hypofunction of this axis, may be related to aggression and CSO through epigenetic changes in the prefrontal cortex [31, 32]. In our sample Kruger et al. [37] found no cortisol reduction in CSO, but it is not unlikely that this may be due to methodological limitations, such as measuring at different times, despite cortisol levels fluctuating throughout the day.

Voxel-based morphometry studies are less sensitive for small structures, such as the hypothalamus [66]. For this reason, this analyses of this area [23–26] may not have yielded results. Additionally, except for Schiffer et al. [25], the field strengths of the MRIs were lower in the mentioned studies and the sample sizes were smaller.

### No significant differences in non-offending people with pedophilia

In contrast to the results regarding CSO, we found no significant difference in exploratory comparisons of left or right hypothalamic volumes between non-offending pedophilic men and the control group. This was in line with our expectations. Differences remained non-significant even with correction for ICV and/or age. This suggests an unchanged hypothalamic macrostructure in non-offending people with pedophilia. Interestingly, there was no gradually progressive increase in hypothalamic volume between the groups (e.g. P + CSO < P-CSO < controls), not even descriptively. Thus, the P-CSO group does not appear to be an intermediate stage between pedophilic offenders and the control group. Contrary to our conclusions previous studies attributed structural changes of the hypothalamus or other brain regions to pedophilia [22, 23, 26]. It can be speculated that their results were influenced by sexual offenders.

### General assessments of the observed hypothalamus volumes

The bilateral hypothalamus volumes measured in vivo with a total mean of 1543 mm^3^ (*SD* = 146.6 mm^3^) are slightly higher than the volumes previously measured with the same method (1427 mm^3^ to 1478 mm^3^) [52]. However, this was to be expected, as our sample consisted exclusively of men and a sexual dimorphism of the hypothalamus, postulated to be larger in men, has been shown before [67–71]. Furthermore, the correlations of hypothalamic volume with age and ICV are consistent with previous reports [66, 67, 72, 73]. The P + CSO group also had the lowest ICV. This finding has to be questioned in future studies by exploring several distinct brain regions involved in control of behaviour and sexual functioning.

### Strengths and limitations

Probably one of the greatest strengths of this study is the distinction between offenders and non-offenders and thus also the fundamental distinction between offenders and people with pedophilia. A disadvantage is that the classification regarding the offender status was necessarily dependent on the self-reporting of the participants, which risks a probability of false statements in, theoretically, both directions. Attempts have been made to mitigate this by ensuring anonymity. Balancing too many or too few exclusion criteria is difficult as excluding individuals with specific diagnoses or medications increases homogeneity at the cost of generalizability. The distribution of lifetime mental and personality disorder diagnoses was significantly different between all groups and the hypothalamic volume may be affected by these. Violent crimes other than CSO, which were not exclusion criteria, may have influenced the results. Even though the sample size is large compared to previous studies in the subject area, it is not sufficient to calculate an equivalence test [64] between the hypothalamus volumes of pedophilic non-offenders and the control group. Since the study is designed cross-sectionally, no conclusions about cause and effect can be drawn. Unbalanced sample distribution among different locations and thus scanner models may have an effect on the GM-TPMs. The evaluated 3-Tesla MR images provided a strong basis for measurements of the brain structures, but a higher field strength would reduce the partial volume effect. Manual segmentation at submillimeter resolution is the most accurate method for this, which is unfortunately hardly feasible for higher case numbers such as in our study due to high time expenditure.

Our large-scale multicenter sample consisted of only men; therefore we can only draw conclusions about males. However, men are most relevant for the research on sexual violence against children, as they are the major group of offenders [74]. To answer whether the results are not only valid for people with pedophilia who committed CSO, but also for CSO in general, exploring non-pedophilic CSO subjects is needed. The structural analysis was based on group comparisons and cannot serve as a basis for a diagnostic criterion or to draw reliable conclusions about individuals.

To our knowledge, no other study has accurately investigated the structure of the hypothalamus in pedophilia with or without CSO. In addition to theoretical considerations about understanding of the neurobiological underpinnings of CSO in pedophilia, the study may add to additional impact in the future, as trait markers of risk factors for committing CSO are needed to stimulate early in the clinical course specific sexual therapeutic treatment which covers more than general psychotherapeutic intervention. The present results need to be replicated in further studies and assessed in relation to, for example, endocrinological and behavioral functions before practical implication can be raised. Implementing an equivalence test would be an important challenge for future studies to discuss the hypothesis of similar brain structures in pedophilic non-offenders and subjects of a control group. However, the required sample sizes for the populations of interest are difficult to realize. Further studies are warranted using functional brain imaging to investigate emotional processing according to the development of pedophilia or CSO over the lifespan. Another interesting question to examine is whether the hypothalamic volume reduction can also be found in a (large) non-pedophilic CSO group to clarify whether our results apply explicitly to the P + CSO group or are valid for CSO exclusively.

The topics of pedophilia and CSO are undeniably emotionally charged. Research such as ours not only provides a better understanding of neural mechanisms underlying pedophilia and CSO, but also contributes to education and public discussion about these matters, rather than reinforcing threats to child welfare with silence and stigmatization.

## Conclusion

Based on hypothalamic volume, we were able to demonstrate a neurobiological signature of sexual violence against children in people with pedophilia, but not of pedophilia per se. The distinction between pedophilia and CSO, which is likewise socially relevant, thus receives further scientific evidence.

## Supplementary Information

Below is the link to the electronic supplementary material.

Supplementary file1 (DOCX 18 KB)

## Data Availability

Research data are not shared. This is because the participants did not consent for open data sharing.
