# Supplementary material for: Hypothalamic volume in pedophilia with or without child sexual offense
Source: Eur Arch Psychiatry Clin Neurosci. 2022 Nov 12;273(6):1295–306. doi: 10.1007/s00406-022-01501-w (PMC10449687; doi:10.1007/s00406-022-01501-w)
Supplement: Supplementary file 1 — Supplementary file1 (DOCX 18 KB) [file 406_2022_1501_MOESM1_ESM.docx]

**Supplementary material**

**Table S1**

*Exploratory comparisons of P-CSO versus control group*

| ***n*** | **Absolute volumes** | | **Corrected for ICV** | | **Corrected for age** | | **Corrected for ICV and age** | |
| --- | --- | --- | --- | --- | --- | --- | --- | --- |
|  | **Left**  **(*M*_Diff_, [95%-CI])** | **Right**  **(*M*_Diff_, [95%-CI])** | **Left**  **(*M*_Diff_, [95%-CI])** | **Right**  **(*M*_Diff_, [95%-CI])** | **Left**  **(*M*_Diff_, [95%-CI])** | **Right**  **(*M*_Dif_, [95%-CI])** | **Left**  **(*M*_Diff_, [95%-CI])** | **Right**  **(*M*_Diff_, [95%-CI])** |
| 206 | 2.57, [‑20.22, 25.36], *p* = .824 | 12.18, [‑9.25, 33.60], *p* = .528 | 2.46, [-17.66, 22.58], *p* = .810 | 12.08, [-7.19, 31.34], *p* = .436 | 5.80, [-16.20, 27.80], *p* = .604 | 14.86, [-5.86, 35.77], *p* = .316 | NA | NA |
| 205 ^OE^ | 3.18, [-19.08, 25.45], *p* = .779 | 13.72, [-6.96, 34.39], *p* = .386 | 3.01, [-16.73, 22.75], *p* = 764. | 13.57, [-5.13, 32.27], *p* = .308 | 6.23, [-15.25, 27.70], *p* = .569 | 16.28, [-3.81, 36.36], *p* = .224 | 5.99, [-12.88, 24.85], *p* = .533 | 16.07, [-1.99, 34.14], *p* = .162 |

Exploratory comparisons are reported with degrees of freedom as subscripts. *Abbreviations:* ICV = intracranial volume, *M*_Diff_ = mean difference, *n* = number of participants, NA = not applicable, OE = statistical outlier excluded, P-CSO = non-offending people with pedophilia, 95%‑CI = 95% Confidence Interval for Difference
